# Formal auditory training in elderly hearing aid users

**DOI:** 10.1016/S1808-8694(15)30154-3

**Published:** 2015-10-18

**Authors:** Elisiane Crestani de Miranda, Daniela Gil, Maria Cecília Martinelli Iório

**Affiliations:** a MSc in Human Communication Disorders - UNIFESP/EPM (Federal University of São Paulo School of Medicine), Speech and Hearing Therapist - Núcleo Integrado de Atendimento, Pesquisa e Ensino em Audição - UNIFESP/EPM; b PhD in Human Communication Disorders - UNIFESP/EPM, Adjunct Professor - Department of Speech and Hearing Therapy - UNIFESP; c Associate Professor - UNIFESP, Adjunct Professor - Department of Speech and Hearing Therapy - UNIFESP. Universidade Federal de São Paulo - UNIFESP

**Keywords:** hearing aids, aged, rehabilitation, training

## Abstract

It is of paramount importance to install hearing rehabilitation programs for the elderly. **Aim**: to check the efficacy of a forma auditory training program for elderly patients fitted with hearing aids for at least three months, by means of speech recognition tests and self-assessment questionnaires. **Methods**: longitudinal contemporary cohort study. We selected 13 elderly hearing aid users, using intra-canal hearing aids in both ears, from both genders, with average age of 65.3 years. This group was randomly divided in Experiment Group and Control Group. The Experiment Group underwent seven formal auditory training sessions, aiming at stimulating their hearing skills for hearing closing, memory, attention, background figure and binaural integration. The participants were assessed by three behavioral tests and one self assessment questionnaire. **Results**: The elderly from the Experiment Group had significantly better performance in the assessments after auditory training in comparison to the Control Group. **Conclusion**: the formal auditory training program in a soundproof booth, associated with the use of hearing aids, improves speech recognition performance and reduces the perception of auditory handicap for the elderly who use intra-canal hearing aids.

## INTRODUCTION

With the progress and improvements achieved in medical technology, there has been an increase in the longevity of the population, and consequently a higher incidence of organic, functional and psychosocial disorders that affect this group. Among them, hearing deterioration is one of the chronic conditions that affect the physical health in aging. Presbycusis is hearing loss caused by aging^1^.

In the hearing rehabilitation of the elderly with presbycusis it is recommended to use hearing aids. With the progress in technology, hearing aids came to the market as increasingly more sophisticated resources, which aim at supplying the needs of the hearing impaired. Nonetheless, some senior citizens can not adapt to the hearing aids and decide against using it or decide for a unilateral fitting, even when they have bilateral hearing loss. The authors also report that a central hearing impairment can negatively impact the hearing aid selection and fitting process^2^.

There are good reasons for believing in physiological changes in the central auditory system, stemming from its stimulation after fitting the hearing aids^3^.

Hearing rehabilitation training programs are used to improve the communication skills of the hearing impaired. There is some evidence that formal hearing training can be efficient in the hearing challenged elderly with sensorineural hearing loss as the one found in most of the people in this age range^3^, ^4^, ^5^, ^6^, ^7^, ^8^, ^9^, ^10^.

It is believed that neural plasticity is not lost with aging. This is an important report, since most users of hearing aids are elderly^11^.

Despite the large number of studies in the area of neural plasticity in the last 15 years, there is still much to study regarding the changes that happen in speech recognition skills because of the reintroduction of the acoustic signal, and if the change magnitude of these changes can be improved with formal hearing training.

Based on these concerns, the goal of the present study is to check the efficacy of the formal auditory training program in elderly citizens with mild to moderately severe bilateral sensorineural hearing loss, users of intracanal hearing aids after three months of their use, by means of speech recognition tests and self-assessment questionnaires.

## METHODS

This study is of a prospective type, clinical and Experiment, and was approved by the Research Ethics Committee of our institution under protocol # CEP 1801/06.

All the participants were informed about the objectives and the methodology of the study proposed and agreed on the performance of the necessary procedures for the study execution, and signed a Free and Informed Consent Form.

For sample selection purposes, the following eligibility criteria were established:

- Having mild to moderately severe bilateral sensorineural hearing loss (hearing threshold mean values of 26 to 70 dB at the frequencies of 500, 1,000 and 2,000 Hz)12.
- Having symmetrical hearing loss of flat or slightly descending curve shape in the high frequencies;
- Having speech recognition index equal to or above 72% bilaterally;
- Having received a hearing aid donation in the last three months; use or have the indication to use intracanal hearing aids in binaural fitting;
- Not having perceivable cognitive alteration or speech alteration;
- Have more than 60 years of age.

Based on the eligibility criteria previously established, we selected elderly from the files of the Núcleo Integrado de Assistência, Ensino e Pesquisa em Audição - NIAPEA, who were fitted with a hearing aid in the year of 2005 and the first quarter of 2006. We found 54 individuals whose medical charts were analyzed in order to check to see if they matched the inclusion criteria. We excluded 40 individuals from the sample, 21 with speech recognition below 72%; nine of them were not found, eight participated in another study that involved hearing training and two were unwilling to take part in the study. Thus, from the initial sample, we had 14 elderly left. Nonetheless, one patient dropped out of the study because of health problems. Then, we had a final sample with 13 elderly, nine women and four men, at an age range varying between 60 and 74 years (mean: 65.30 years).

All the participants were submitted to evaluation only after they were assigned to the Experiment Group (those submitted to hearing training) and to the Control Group (those who were not submitted to hearing training), and the individuals themselves pick a number to be randomized to which group they would be sent to. Thus, the Experiment Group (EG) had six individuals and the Control Group (CG) had seven.

The elderly who were interested in participating in the Hearing Training Program, but because of this random assigning were included in the Control Group, were invited to participate in it after the end of the study.

All assessment procedures, as well as the auditory training were carried out with the elderly using their intracanal hearing aid.

The participants in the study were submitted to: Tonal Audiometry with the hearing aid and with ear phones, signal/noise ratio test obtained from the Recognition of Phrases in Noise (SRPN), non-sensitized Speech Recognition Index test (SRI with recording), Speech Test with White Noise (STWN) and Hearing Handicap Inventory for the Elderly - HHIE. The assessment duration time was of approximately 45 minutes.

The group that received hearing training (Experiment Group) was evaluated in three different times, before starting the training (1st assessment), after eight weeks of hearing training (8th week / 2nd assessment) and four weeks after the end of treatment (12th week/3rd assessment). The group that did not receive training, called Control Group went through the same evaluation system of the Experiment Group, following the same time intervals between the evaluations, which were carried out by calling the person in.

In order to characterize a double blind study, the reevaluations were carried out by a different examiner from the one that made the first assessment, who did not know whether or not the individual had been trained.

Formal Hearing Training (FHT) proposed in this study was organized in seven sessions, held once a week, with duration of 50 minutes each^10^, ^13^. The material used to hold the hearing training were the CDs that were part of the “Central Auditory Processing: Assessment Manual” book^14^.

**Figure 1:**
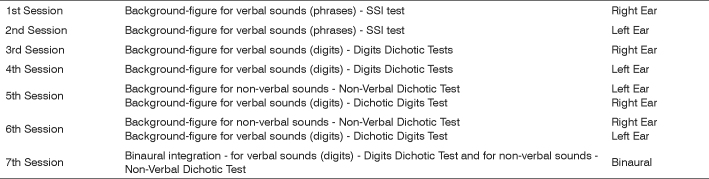
The hearing training schedule and the activities proposed in each of them were as follows:

Hearing training was carried out with earphones, thus, it was possible to train the right and left ears, separately. We established a fixed intensity for stimulus presentation (verbal and non-verbal) and we varied the competitive noise intensity (Speech Noise). From the first to the sixth training sessions the patients had single ear hearing tasks in the presence of a competitive noise, varying the signal/noise ratio from positive (more favorable) to negative (less favorable). The last training session involved a binaural integration task (Figure 1).

## RESULTS

In all the tests and evaluations carried out, the differences between the right and left ears were not statistically significant, in other words, the ear variable is not a difference causing factor for the tests performed. Thus, in the analyses presented below we considered both ears, in such a way that the sample size doubled, providing more reliability to the results analyses.

For the statistical analyses of this study we used the ANOVA Averages Comparison test and the Tukey Multiple Comparisons test. We established a 0.05 (5%) level of significance and the confidence intervals built along the study were of 95% of statistical confidence.

Initially, we studied the effect of hearing training among the evaluations according to the Group (Experiment and Control). We compared the values among the three evaluations. On Tables 1, 2, 3 and 4 we see the descriptive measures of SRPI, SWWN, N/S ratio - PRTN and HHIE values respectively and the comparative study of the 1st, 2nd and 3rd evaluations in both groups.

**Table 1:** SRPI descriptive measures on the 1st, 2nd and 3rd evaluations obtained in Groups E and C, and results from the ANOVA (p-value) test.

SRPI	CG (without training)			EG (with training)		
	Asses. 1	Asses. 2	Asses. 3	Asses. 1	Asses. 2	Asses. 3
Mean	79,14	78,00	79,43	82,67	85,33	87,00
Median	80	76	80	82	88	88
Standard deviation	5,91	6,42	6,05	7,50	7,50	5,43

p-value		0,378			0,013*	

SRPI - Speech Recognition Perceptive Index; EG - Experiment Group; CG - Control Group

* Significant p-value < 0.05 (5%)

**Table 2:** SWWN test descriptive measures for the 1st, 2nd and 3rd evaluations obtained for Groups C and E, and the results from the ANOVA test (p-value).

SWWN	CG (without training)			EG (with training)		
	Asses. 1	Asses. 2	Asses. 3	Asses. 1	Asses. 2	Asses. 3
Mean	67,43	65,29	67,00	69,33	81,67	82,33
Median	70	70	72	76	80	80
Standard deviation	15,20	14,83	14,40	18,32	9,26	6,71

p-value		0,164			0,001*	

Legend: SWWN - Speech With White Noise; EG - Experiment Group; CG - Control Group

* significant p-value < 0.05 (5%)

**Table 3:** Descriptive measures of the S/N ration in the PRTN test for the 1st, 2nd and 3rd assessments of Groups C and E, and results of the ANOVA test (p-value).

PRTN (S/N ratio)	CG (without training)			EG (with training)		
	Asses. 1	Asses. 2	Asses. 3	Asses. 1	Asses. 2	Asses. 3
Mean	3,78	5,38	5,68	5,22	3,01	2,95
Median	3	5	5	6	3	3
Standard Deviation	2,43	3,03	2,93	3,19	1,85	2,26

p-value		0,001*			0,005*	

Legend: PRTN - Phrase Recognition Threshold in Noise; S/N - Signal to Noise ratio; EG - Experiment Group; CG - Control Group

* Significant p-value < 0.05 (5%)

**Table 4:** HHIE descriptive measures in the 1st, 2nd and 3rd assessments of Groups C and E and the results from the ANOVA test (p-value).

HHIE	CG (without training)			EG (with training)		
	Asses. 1	Asses. 2	Asses. 3	Asses. 1	Asses. 2	Asses. 3
Mean	21,43	22,57	20,57	55,67	21,33	12,33
Median	12	12	6	57	19	9
Standard deviation	26,92	33,32	33,40	32,82	14,40	10,61

p-value		0,840			0,009*	

Legend: HHIE - Hearing Handicap Inventory for the Elderly; 1 - 1st assessment; 2 - 2nd assessment; 3 - 3rd assessment; EG - Experiment Group; CG - Control Group; Asses. - Assessment

* Significant p-value < 0.05 (5%)

We noticed that there was a significant difference among the results obtained in the three assessments in all the Experiment Group tests. In order to investigate exactly where the difference occurred we used the Tukey Multiple Comparisons Test and its results can be seen on Table 5.

**Table 5:** Results from the Tukey Multiple Comparisons Test (p-values) for SRPI, SWWN, PRTN and HHIE from the Experiment Group.

EG (with training)		1st Assessment	2nd Assessment
SRPI	2nd Assessment	0,013*	
	3rd Assessment	0,020*	0,295
SWWN	2nd Assessment	0,002*	
	3rd Assessment	0,007*	0,586
PRTN - S/N	2nd Assessment	0,006*	
	3rd Assessment	0,001*	0,899
HHIE	2nd Assessment	0,036*	
	3rd Assessment	0,022*	0,152

Legend: SRPI Speech Recognition Perceptive Index; Speech With White Noise; PRTN Phrases Recognition Threshold in Noise; S/N - Signal/Noise ratio; HHIE - Hearing Handicap Inventory for the Elderly; EG - Experiment Group

* Significant p-value < 0.05 (5%)

On the second stage, we investigated whether the hearing training had been effective by comparing the results obtained from the elderly who were trained (EG) and those who were not (CG). It is presented on Tables 6, 7, 8 and 9 the comparative study between Groups C and E for the SRPI, SWWN, PRTN, S/N ratio and HHIE questionnaire.

**Table 6:** SRPI descriptive measures in the elderly from the C and E Groups, and results from the ANOVA (p-value) test.

	SRPI (1)		SRPI (2)		SRPI (3)	
	CG	EG	CG	EG	CG	EG
Mean	79,14	82,67	78,00	85,33	79,43	87,00
Median	80	82	76	88	80	88
Standard Deviation	5,91	7,50	6,42	7,50	6,05	5,43

p-value	0,193		0,013*		0,003*	

Legend: SRPI - Speech Recognition Perceptive Index; 1 - 1st assessment; 2 - 2nd Assessment; 3 - 3rd assessment; EG - Experiment Group; CG - Control Group

* Significant p-value < 0.05 (5%)

**Table 7:** Descriptive measures of the SWWN test obtained from the elderly in Groups C and E by assessment and results from the ANOVA (p-value) test.

	SWWN (1)		SWWN (2)		SWWN (3)	
	CG	EG	CG	EG	CG	EG
Mean	67,43	69,33	65,29	81,67	67,00	82,33
Median	70	76	70	80	72	80
Standard Deviation	15,20	18,32	14,83	9,26	14,40	6,71

p-value	0,774		0,003*		0,002*	

Legend: SWWN - Speech With White Noise; EG - Experiment Group; CG - Control Group; 1 -1st assessment; 2 -2nd assessment; 3 - 3rd assessment

* Significant p-value < 0.05 (5%)

**Table 8:** Descriptive measures of the S/N ratios of the PRTN test obtained from the elderly in Groups C and E by evaluation, and results from the ANOVA (p-value) test.

	PRNT (1)		PRNT (2)		PRNT (3)	
	CG	EG	CG	EG	CG	EG
Mean	3,78	5,22	5,38	3,01	5,68	2,95
Median	3	6	5	3	5	3
Standard deviation	2,43	3,19	3,03	1,85	2,93	2,26

p-value	0,202		0,027*		0,015*	

Legend 1: PRTN - Phrases Recognition Threshold in Noise; S/N - Signal to Noise ratio; EG - Experiment Group; CG - Control Group; 1 -1st assessment; 2 -2nd assessment; 3 - 3rd assessment

* significant p-value < 0.05 (5%)

**Table 9:** Descriptive measures from HHIE results obtained from the elderly in Groups C and E per assessment, and results from the ANOVA test (p-value).

	HHIE (1)		HHIE (2)		HHIE (3)	
	CG	EG	CG	EG	CG	EG
Mean	23,00	55,67	23,67	21,33	21,33	12,33
Median	12	57	8	19	3	9
Standard deviation	29,14	32,82	36,36	14,40	36,52	10,61

p-value	0,046*		0,900		0,626	

Legend 1: HHIE - Hearing Handicap Inventory for the Elderly; EG - Experiment Group; CG - Control Group; 1 -1st assessment; 2 -2nd assessment; 3 - 3rd assessment

* Significant p-value < 0.05 (5%)

## DISCUSSION

In the first stage of this study we assessed the effect of hearing training between evaluations, per group, which revealed that the elderly in the EG - in the evaluation carried out before hearing training (1st evaluation), had a regular performance in the SRPI test (82.67%) and as predicted, they got worse when the competitive noise was introduced (69.33%).

After Formal Hearing Training (FHT) we observed a significant improvement in the results obtained from both tests (SRPI and SWWN), having the scores within normal limits, 87% and 82.33%, respectively (Tables 1 and 2).

The same has been observed in the S/N ratio in the speech recognition perceptive index in terms of an improvement in the patients” performance after FHT (5.22 to 2.58). Nonetheless, results after hearing training did not reach the values standardized for young adults with hearing loss. (Table 3).

These data show that the hearing training in a sound-treated booth improves the speech recognition skills in favorable and adverse hearing settings.

The subjective assessment carried out by means of a self-assessment HHIE questionnaire revealed a significant reduction of self-perception in the auditory handicap in the assessments after hearing training (from 55.67% to 21.33% and 12.33%), and the final results showed percentages below 16%, which characterizes a non-perception of the auditory handicap, in other words, the hearing and non-hearing difficulties generated by the hearing impairment are not perceived (Table 4).

Through hearing rehabilitation programs, it is possible to reduce the very perception of the hearing handicap in the elderly population which will reflect on an improved quality of life, promoting social contact and reducing isolation of people^15^.

Besides the study held to investigate the effect of hearing training (1st X 2nd assessments) we carried out an assessment one month after the end of treatment (3rd assessment), in order to check if the improvements obtained would keep throughout time. In this study, we did not see differences between the results from the 2nd and 3rd assessments, although a mild improvement has been seen in the last assessment (Table 5).

Neural plasticity is not lost with aging^3^, ^11^. The central auditory nervous system of the elderly is capable of changing and, with hearing training; it is possible to improve speech recognition skills, especially in a noisy background^4^.

Auditory plasticity has the most significant role in hearing training^16^. Numerous studies show that auditory pathways function within the Central Nervous System (CNS) can be improved with hearing training^17^, ^18^, ^19^.

It is believed that once the neural substrate has been changed and the behavioral pattern has been learned at the end of the training sessions, the individual goes back to his/her daily routines, the environment itself, with all its demands, reinforce the pattern learned and maintains the trend towards improvement^10^.

The amplification provided by the hearing aid does not maintain the same sound reception quality in silence or in noisy settings. This happens because the hearing aid solves the problem of sensitivity loss, but not the problems associated with speech detection and understanding in the presence of background noise. Nonetheless, the elderly in the EG showed that communication difficulties they had to face in their daily routines, were minimized with the association of hearing aids and hearing training.

Based on the results found among the elderly from the Experiment Group (EG), we could notice that both the behavioral tests and the subjective assessments were improved after hearing training in a sound-treated booth, in other words, the FHT brought about the development of speech recognition skills (words and phrases) and auditory closure, as well as a reduction in the hearing handicap.

These data indicate that the elderly with hearing loss are capable of significantly improve their capacity to recognize speech and reduce the impairments created by their hearing loss by means of an auditory training. These findings agree with those from similar studies found in the literature^9^, ^20^.

In comparing the results between the Group Control assessments, speech tests show that mean SRPI and SWWN values were kept the same throughout the period studied (Tables 1 and 2). Nonetheless, the skill to recognize phrases in a noisy background showed a significant worsening in the final evaluations (2nd and 3rd assessment) of this study (Table 3).

Thus, we have noticed that the simple fitting of a hearing aid does not produce the ideal adaptation for the auditory system and the consequent improvement in speech skills^7^, once the results are stable throughout a period of 12 weeks.

To recover normal hearing with a hearing aid may seem to be an impossible goal for some patients; however, normal hearing does not mean not having difficulties in certain situations. When there is hair cell damage, even reaching this goal, supraliminal deficits may not compensate, as for example, frequency and temporal resolution, which impair their performance in adverse hearing environments^21^. In the elderly of the CG, the hearing thresholds with hearing aids showed satisfactory results; however, it did not match the speech recognition skill.

The hearing handicap self-perception results of the Control Group elderly did not show significant changes in 12 weeks. Thus, the use of hearing aids did not change the handicap self-perception along time (Table 4).

These findings are similar to the ones seen in this study^22^, which followed the elderly patients up after hearing aid fitting for approximately two years, also did not observe changes in the hearing handicap during the period studied.

Then, we can think that not having the hearing training could have contributed to the worsening in the skill assessed, in agreement with some studies described in the literature, in whom the auditory training was not carried out^23^, ^24^, ^25^.

In the second data analysis, we studied the effects of hearing training between the groups (CG X EG). By analyzing the results from the SRPI, SWWN and S/N ratio - PRTN test, we noticed that the elderly from the Experiment Group (EG) showed a better performance as far as speech recognition is concerned with and without noise, in contrast to the Control Group (CG) in the assessments after hearing training (2nd and 3rd assessments - Table 6, Table 7, Table 8).

These data are in agreement with the ones found in a similar study^10^, which also stated a significant difference between the trained (Experiment) and untrained (Control) people in the SRPI and SWWN tests after FHT.

The pure and simple fitting of sound amplification does not consider the need of many hearing impaired people. Sound amplification facilitates reception, but does not improve the other conversational components - the listening, the understanding and the communication, aspects that can be worked upon during hearing training^26^.

The use of hearing aids must include some type of hearing training, either formal or informal, in order to teach the patient to deal with this new amplified sound and, even more important, the new sound processing pattern^17^, ^27^. For the elderly it is extremely important to develop an auditory training in noisy environments, because the most common complaint among these patients is the very difficulty they have with speech understanding in environments where hearing is difficult^28^.

Regarding the results obtained in the assessment of the auditory handicap by means of the HHIE questionnaire, in the beginning of the study (1st assessment) the elderly from the experiment and control groups were significantly different in relation to the perception they had of the hearing loss, regarding social and emotional aspects. Despite the fact that the elderly were divided in random groups by lottery, the EG elderly had a higher handicap, severe (55.67%), while the elderly from the CG of mild to moderate level (23%) in the first assessment (Table 9).

It”s worth stressing that the Experiment Group elderly, submitted to auditory training, were more active (had a professional activity, attended English classes, participated in choirs and social meetings) than the elderly from the Control Group. We can assume that these elderly, since they are more active (EG) were more needed in communication situations and thus had a more acute perception of the negative effects of their hearing loss, as well as the limitations of sound amplification in adverse hearing environments, and consequently, this reflected in a higher degree of dissatisfaction.

Nonetheless, this difference was not maintained after FHT, and in the last assessment (3rd assessment) the elderly submitted to training presented mean percentages that characterized the non-perception of the auditory handicap (12.33%), while the elderly from the Experiment Group (EG) remained with mild to moderate perception (21.33%). These findings suggest the influence of a FHT as well as a direction towards the reduction of self-perception of the difficulties caused by the hearing loss, consequently providing a quality improvement in the lives of these elderly, and also a better use of their hearing aids.

Handicap variation reflects the individual differences in perception of negative effects of the hearing loss and it is also known that this disadvantage is also influenced by their life styles in their communities^29^. The auditory handicap self-assessment can be influenced by non-audiologic factors, such as race, gender, age, personality, social support, as well as socio-economic factor, health status and attitudes regarding the use of hearing prosthesis, amongst others^30^. Therefore, all these factors can also interfere in the success of the hearing aid fitting and certainly in the hearing impairment in the life of the patient.

## CONCLUSION

The hearing training program in a sound-treated booth, associated with the use of hearing aids, improves the user”s performance in their speech recognition skills and reduces the auditory handicap of the elderly who use intracanal hearing aids.
